# The Environmental Hazards and Treatment of Ship’s Domestic Sewage

**DOI:** 10.3390/toxics12110826

**Published:** 2024-11-19

**Authors:** Yanan Zhang, Bensen Xian, Wenkai Sun, Ruifang Lu, Qin Zhang, Mei Wang, Dandan Xu, Huili Liu, Shaoyuan Bai, Mingming Fu

**Affiliations:** 1 The Guangxi Key Laboratory of Theory and Technology for Environmental Pollution Control, Guilin University of Technology, Guilin 541004, China; zyanan@glut.edu.cn (Y.Z.); xianbensen@glut.edu.cn (B.X.); 18837492300@163.com (W.S.); ruifanglu@glut.edu.cn (R.L.); qinzhanggl@163.com (Q.Z.); liuhuili@glut.edu.cn (H.L.); baisy@glut.edu.cn (S.B.); 2 Collaborative Innovation Center for Water Pollution Control and Water Safety in Karst Area, Guilin University of Technology, Guilin 541004, China; xudandan@glut.edu.cn; 3 Hengsheng Water Environment Treatment Co., Ltd., Guilin 541100, China; wm905242022@163.com; 4 Key Laboratory of Carbon Emission and Pollutant Collaborative Control, Education Department of Guangxi Zhuang Autonomous Region, Guilin University of Technology, Guilin 541004, China; 5 College of Environmental Science and Engineering, Guilin University of Technology, Guilin 541004, China

**Keywords:** domestic sewage from ships, environmental hazards, treatment methods, MBR

## Abstract

With the rapid development of the modern shipping field, the damage caused by ship pollution to the global inland waterways and marine ecosystems has attracted extensive attention from the international community. However, there are fewer reviews on the environmental hazards of domestic ship sewage and its treatment, and a systematic summary of the environmental hazards posed by ship domestic sewage and its treatment is lacking. Based on summarizing the various environmental hazards brought about by a ship’s domestic sewage and the corresponding treatment methods, this study elaborates, in detail, on the specific hazards of the main toxic and hazardous substances contained in a ship’s domestic sewage on the environment and organisms, and the treatment methods of the ship’s domestic sewage and their treatment effects, such as membrane bioreactor (MBR). It is also pointed out that MBR has great potential in the direction of ship domestic sewage treatment, and the solution of its membrane pollution and other problems as well as the exploration of the combination of MBR and other treatment methods will become the focus of future research. A theoretical reference is provided for the study of environmental problems caused by domestic sewage from ships and their treatment options.

## 1. Introduction

A ship is a transport vehicle used for traveling or docking on water for the carriage of goods, passengers, or operations. Inland waterway transport ships are ships that use ships to transport passengers and goods on rivers, streams, lakes, reservoirs, or artificial waterways and can be divided into inland waterway passenger ships and inland waterway cargo ships [1]. Marine transport ships are ships engaged in the transport of passengers and goods in open waters on the oceans and can be divided into seagoing passenger ships and seagoing cargo ships [2].

**Figure 1:**
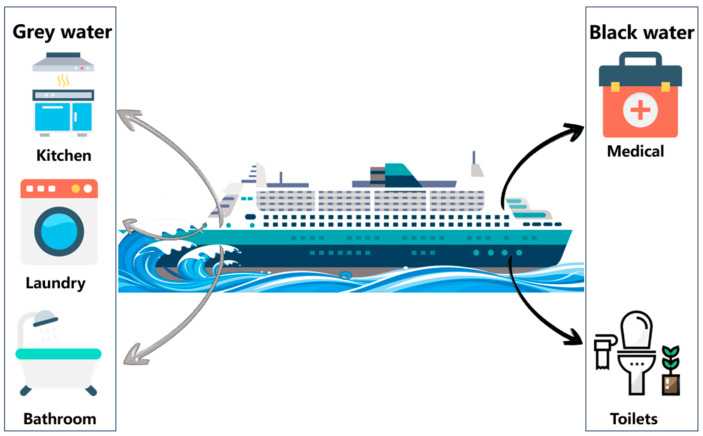
Main sources of domestic sewage from ships.

However, the transport time of various types of ships varies; some ships take one hour for a trip, while others may take several days or months, which means that a lot of domestic sewage will be generated on board the ships. Domestic sewage from ships is sewage generated by members of the crew and passengers living on board, including all forms of commode discharges and some rubbish; discharges from hand-washing sinks, bathtubs, and drainage outlets in medical offices or drug storage rooms; feces from livestock premises; and other sewage mixed with the above-mentioned emissions or wastes [3]. Depending on the quality of the water discharged, it could be classified as “black water” or “grey water” (as shown in Figure 1). “Black water” refers to drainage from public toilets and medical rooms where the concentration of domestic sewage is high, i.e., fecal sewage and medical sewage; “grey water” refers to kitchen “grey water” (domestic sewage discharged from kitchens, canteens, dishwashing rooms, steaming rooms, etc.) and cleaning “grey water” (sewage discharged from clean rooms, shower rooms, laundry rooms, etc.), which is less polluting to the environment [4,5].

To solve environmental problems posed by ship domestic sewage, researchers have designed various feasible ways to treat ship domestic sewage. The latest research shows that the membrane bioreactor (MBR) is a popular research direction in the field of ship domestic sewage treatment, and researchers have designed a variety of MBR-based ship domestic sewage treatment units by improving it [6,7]. At the same time, other methods are also being developed for ships to provide more possible ways to treat domestic sewage. To investigate the environmental hazards of domestic sewage from ships and the treatment effectiveness of existing sewage treatment devices and their future development direction, this study reviews the hazards of shipping domestic sewage for the environment and summarizes the treatment of ship domestic sewage and its specific treatment effect. This review can provide a reference for the government’s Maritime Administration to set the standard of ship domestic sewage treatment and provide guidance for environmental agencies to produce and research ship domestic sewage devices and shipowners to choose and purchase ship domestic sewage devices. It also allows researchers to learn more quickly about the progress in the field of marine sewage treatment.

## 2. Pollutants in Ships’ Domestic Sewage and Their Hazards

Domestic sewage generated on board ships differs from domestic sewage in urban areas because the sanitation system on board ships has a shorter discharge time, so the waste discharged is less decomposed than in urban drainage [8]. In addition, due to the difference in water consumption between sanitary appliances such as toilets used on board ships and those used by the general population, water production on board is difficult. There is often a need to conserve water, usually by using a small amount of water for flushing the toilet, which results in shipboard domestic sewage containing greater concentrations of environmental pollutants such as BOD and suspended solids compared to urban domestic sewage (as shown in Table 1). It is, therefore, much more polluting [9]. Previous studies have shown that a ship’s domestic sewage is characterized by high ammonia nitrogen, high COD, high BOD, etc., and often contains microplastics, heavy metals, pharmaceutical compounds, organotin, and other substances that are toxic and harmful to the environment. If ship domestic sewage is directly released into the waters in which it is found, it can cause serious environmental pollution and threaten aquatic ecosystems as well as human health.

In the process of ship domestic sewage generated in the operation of the phenomenon of wanton discharge, it is easy to cause serious pollution of water resources [18]. Ship domestic sewage for the river, lake, and seawater environment is very obvious, in addition to directly leading to the destruction of oxygen-consuming organisms in the water area; also, because feces are high in nutrients such as nitrogen and phosphorus, for waters such as the large lakes, estuaries, and bays, the large-scale influence of domestic pollution from ships will promote the generation of eutrophication in the aquatic environment, thus impairing the self-purification balance of the waters and consequently polluting the water environment of the region [19,20]. The levels of viruses, bacteria, and parasites in untreated ship domestic sewage are also alarmingly high, directly interfering with the sanitary and health standards of the waters and leading to the spread and propagation of diseases [21]. Pollution from ships is characterized by high fluidity, the fluidity of ships determines that the pollutants brought about by them may not be limited to or fixed at one point and stand still, and pollution may spread to many sea areas and regions, causing great inconvenience to management after the event [22].

**Figure 2:**
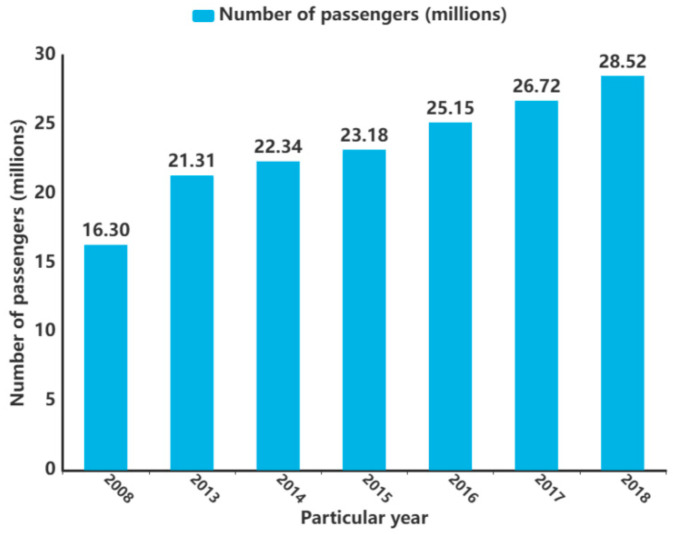
Demand for international cruises (in millions).

Current figures show that, as of 2017, there were over 25.4 million cruise passengers in Europe alone [23]. The rest of the world, too, experienced significant growth (as shown in Figure 2) [24,25]. The remarkable growth of the maritime industry has also presented the world with several serious challenges for the marine environment [26].

### 2.1. Conventional Pollutants in Ships’ Domestic Sewage

Shipborne pollution is one of the main factors contributing to the pollution of marine environments, and with the increase in the number of ships and their passengers, there is an increased risk of various substances contributing to marine pollution, including ship domestic sewage [27,28]. Žarko Koboević et al. analyzed ocean contamination from ship sewage in a study where they sampled the ocean in eight separate coastal areas within a period of fourteen months. The results of the study proved that domestic sewage discharged from small boats and recreational vessels was the cause of pollution of marine coastal waters in the study region [29]. Domestic sewage discharged from ships may contain various mixtures of several hundred to several thousand types of petroleum hydrocarbons (Chrysene 0.05–0.14 μg/L, DDPHs 0.46 μg/L) [30,31]. The discharge into the ocean ecosystem of such pollutants may have adverse effects on the well-being of marine organisms, as the hydrocarbons accumulate in different parts of the food chain, thereby disrupting the biochemical or physiological processes of numerous species [32]. Studies have shown that cruise ships carrying 2000–3000 passengers in the Baltic Sea produce an average of 550–800 tons of grey water and 100–115 tons of black water a day and that domestic sewage discharged from cruise ships causes serious environmental problems such as the eutrophication of water bodies [33]. Releases of such sewage effluents to marine water environments can lead to severe ecological degradation and a range of non-communicable diseases in fish, with high ammonia nitrogen and high-carbon sources of domestic sewage from ships, triggering a variety of bacterial diseases in fish by increasing the proliferation of micro-organisms in the aquatic environment and suppressing the immune defense mechanisms of fish [34]. Excessive nitrogen and phosphorus in domestic sewage discharged from ships can lead to algal blooms that may damage marine ecosystems [35,36]. In the production and operation process of inland waterway vessels, accompanied by the daily life of the crew, a large amount of domestic sewage will be generated, which will be discharged arbitrarily, causing serious harm to the surrounding water environment and the health of coastal residents [37]. Studies have shown that the per capita volume of fecal wastewater for crew members on river cargo ships is 14 L/day. The fecal pollution loadings were 23 g/person/day for BOD_5_ and 28 g/person/day for SS. The fecal sewage pollution concentration BOD_5_ was up to 1656 mg/L; SS was 2016 mg/L [38].

### 2.2. Novel Pollutants in Ships’ Domestic Sewage

Microplastics were found for the first time in grey and black water samples discharged from ships in a study by Renate Kalnina et al., who collected 50 water sample datasets from black and grey water samples from five ocean carriers and showed that the average number of particles isolated was n = 72 per liter in grey water samples and n = 51 per liter in black water samples [39]. Among the six hundred and fourteen isolated microparticles, fibers n = 285 (46.4%) were the commonest, closely preceded by other stiff particles n = 226 (36.8%) as well as soft particles n = 104 (16.8%). Analysis using scanning electron microscopy (SEM) and energy-dispersive X-ray spectroscopy (EDS spectroscopy (EDS)) showed that the surface of these particles contained ecotoxic chemical elements. Plastics in domestic sewage discharged from ships collectively kill hundreds of thousands of marine mammals each year [40,41]. Merchant ships at sea emit 639,000 microplastics per day around the world; thus, ships are a major source of plastic debris [42]. In addition, personal care products and pharmaceutical compounds (PPCPs) have been shown to harm aquatic organisms, and ships at sea are considered to be an important source of pharmaceutical compounds [43]. The German NAUTEK R&D project detected PPCPs discharged from passengers or on-board medical rooms, both in black water and grey water from seagoing vessels (as shown in Table 2), with 16 out of 21 targeted substances being identified and each drug’s peak concentration predominantly occurring in the black water (3.9 μg/L for carbamazepine, 29 μg/L for ibuprofen, and 0.04 μg/L for diclofenac; 0.04 μg/L; 9 μg/L, ibuprofen 29 μg/L, and diclofenac 0.04 μg/L), whereas the grey water was primarily ointment residues, ultraviolet light filters, and flame retardants (peak concentrations of diclofenac and bisphenol A were 0.65 μg/L and 8 μg/L, respectively) [44]. These pharmaceutical compounds may harm aquatic life and human health [45].

### 2.3. Metal Pollutants in Ships’ Domestic Sewage

#### 2.3.1. Organometallic Contaminants

Organotin (OT) complexes like tributyltin (TBT), as well as triphenyltin (TPT), have been extensively utilized as antifouling compounds since the 1960s to provide protection to ships against the attachment of marine organisms [52]. OT compounds, especially coatings containing TBT and TPT, are very effective antifouling agents, preventing fouling of hulls for 3–5 years [53]. Due to their special effect on most fouling and marine organisms, OTs are used extensively in the marine sector and have saved the shipping industry a great deal of money [54].

However, these OT compounds are discharged into the oceans and mixed with various types of ship sewage through ship trafficking, ship dismantling activities, etc. [55,56]. Although the inorganic form of tin (Sn) is considered avirulent, there are complex patterns of toxicology for organic tin compounds [57]. In particular, TBT in unfouling varnishes has been called the highest-toxicity material that has ever been discharged into the ocean environment [58]. TBT is extremely poisonous for marine species, although at a very small concentration [59]. Despite worldwide restrictions on the use of OT, large quantities of OT are still discharged into the aquatic environment, causing serious pollution of water ecosystems [60]. OT compound expression damages the neurological system, influences organismal growth as well as energetic processes, and disrupts internal endocrine systems by causing abnormalities in gonadal development [61]. Moreover, these OTs could potentially react together with other contaminants to lead to a combination of toxicity that can accumulate through the food chain and pose a threat to human life [62]. As a result, the International Maritime Organization (IMO) established a global treaty to ban the use of TBT-based paints from 1 January 2003 and a total ban by 1 January 2008 [63]. Although regulations have reduced TBT levels in the marine environment, vessel cleaning and painting locations can still be potential localized sources of TBT [64]; studies have shown that ship-generated TBT inputs still occur in parts of the global aquatic environment [65].

#### 2.3.2. Heavy Metal Contaminants

**Figure 3:**
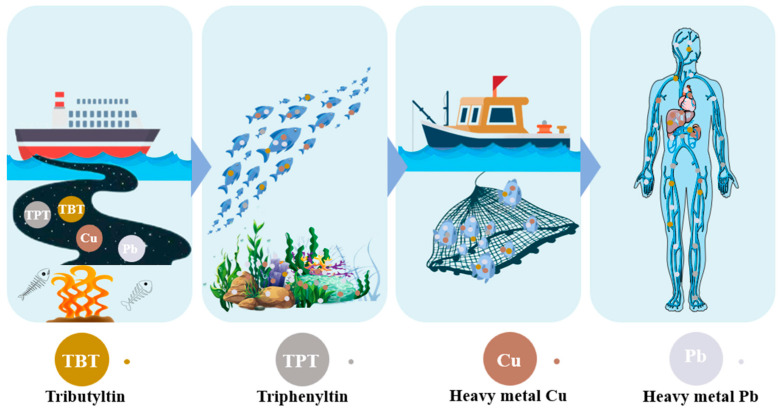
Hazard of metal pollutants in ships’ domestic sewage to organisms.

Heavy metals pose severe risks to the water environment as well as to the safety of aquatic ecosystems due to their difficult degradation and high toxicity, while domestic sewage discharged from shipping and ships is usually considered to be the main source for heavy metals such as Pb and Cu [66,67]. Heavy metals can impact water-living creatures, accumulating in multiple organs, causing adverse effects such as damage to oxidation, disruption of the endocrine system, and suppression of the immunological system [68]. Heavy metals are not biodegradable and, therefore, tend to accumulate in living organisms, endangering people’s well-being via the food chain and drinking water [69,70]. However, these heavy metals, even though at extremely small levels, can cause multi-organ injuries that affect the liver, skin, lungs, prostate, kidneys, stomach, and esophagus and can also lead to degenerative neurological disorders and illnesses [71]. The hazards of metal contaminants to organisms are shown in Figure 3.

## 3. Discharge Standards for Domestic Sewage from Ships

### 3.1. International Standard for Discharge of Domestic Sewage from Ships

As the ocean environment is becoming increasingly polluted, mankind is attaching more and more importance to the conservation of the ocean, and the concept of environmental conservation is becoming stronger and stronger. The IMO developed the MARPOL 73/78 Convention, which provides for the discharge and treatment of domestic sewage in its Annex IV (Code for the Prevention of Pollution from Ships’ Domestic Sewage), which became effective on the 27th September 2003 and established limits for the emission of ship-generated wastes according to the type of wastes and the area of discharge [72,73]. MARPOL Annex IV applies to all ships of 400 gross tons and above on international voyages, and to ships of less than 400 gross tons on international voyages that are authorized and licensed to carry 15 or more persons [74].

Article 11 of MARPOL 73/78, Annex IV, ‘Code of Practice for the Prevention of Pollution by Domestic Sewage from Ships’, clearly defines the discharge of domestic sewage as follows: A ship discharges crushed and sterilized domestic sewage, with equipment authorized by the competent authority, further than 3 n miles from the nearest land, or further than 12 n miles from the nearest land, where the ship discharges domestic sewage without comminution and disinfection beyond 3 n miles from the nearest land or 12 n miles beyond the closest land. In all cases, however, domestic sewage stored in the collection tanks shall not be discharged immediately but shall be discharged at a suitable rate, approved by the competent authority, when the ship is traveling at a speed of not less than 4 kn. If the closest point of land is within 3 n miles, a domestic sewage treatment device approved by the competent authority is required to be used, and the effluent discharged does not create any observable floating solids in the water in its vicinity and does not discolor the water; also, the *Coliform* count must be ≤100 CFU/100 mL, the BOD_5_ must be ≤25 mg/L, and the TSS must be ≤35 mg/L [75].

Within the framework of the IMO Convention, the discharge performance indicators for domestic sewage from ships have mainly undergone three major changes: resolutions MEPC.2(VI), MEPC.159(55), and MEPC.227(64). IMO has continuously raised ship domestic sewage discharge standards in recent years, making them more stringent than those in the past [76].

### 3.2. Discharge Standards for Domestic Sewage from Chinese Ships

China applied IMO in 2006 to accede to the amended Annex IV, which was approved and entered into force on 2 February 2007 in China. After this, China newly promulgated the discharge standard for water pollutants from ships (GB 3552-2018) in 2018 as the latest standards changed by the IMO Convention, with more and more stringent requirements for biochemical oxygen demand (BOD), suspended solids (TSS), *Coliform bacteria*, and pH indicators for ship and offshore platforms’ domestic sewage discharges, as well as a gradual increases in COD, RC, TN, TP and other indicator requirements; this standard has been very positive in the control of pollutant discharges from ships and improvements in the environmental quality of navigable waters in China [10,77]. A comparison of the IMO and Chinese standards for domestic sewage discharge from ships is shown in Table 3.

## 4. Ship’s Domestic Sewage Treatment Methods

Pollution of the world’s water resources is a serious problem, and one of the main reasons for ships polluting water resources is the lack of environmentally friendly equipment required for ships, while there are many difficulties in treating domestic sewage from ships, including ship area constraints, high salinity, high pollutant concentration, power supply for sewage treatment, sludge after sewage treatment, poor stability, etc. (as shown in Table 4). Therefore, it is crucial to develop suitable treatment technologies for domestic sewage from ships and to improve the quality of their treatment [78,79]. This section mainly discusses the domestic sewage treatment methods that have been proven to be feasible for ship domestic sewage; part of the land-based domestic sewage treatment technologies based on physics, chemistry, etc., have not been proven to be used for ship domestic sewage treatment, and are not included in the scope of discussion in this section.

### 4.1. Non-Discharge-Type Ship Domestic Sewage Treatment Methods

Ship domestic sewage treatment methods can be classified as discharge type and non-discharge type according to where the sewage is discharged. The methods in which a ship does not discharge domestic sewage into the water environment during its voyage are all non-dischargeable ship domestic sewage treatment methods [89]. The sewage treatment methods for non-discharge ships mainly involve the collection and storage of sewage, as well as subsequent discharge arrangements. Surveys have shown that small vessels operating on inland waterways are predominantly classified as non-discharge vessels, with their primary distinguishing feature being the incorporation of a sophisticated and highly efficient sewage collection system. By meticulously addressing each phase of the process, this system ensures environmental compliance and safeguards the ecological integrity of inland waterways [90,91]. Ships’ domestic sewage collection devices, such as domestic sewage storage compartments (cabinets), will be calculated according to the operating conditions, persons on board, days of accommodation, and other relevant factors; if the volume is sufficient to store domestic sewage generated by the ship, domestic sewage that is not treated on board the ship after the sewage cabinets are full of domestic sewage will be accepted and treated by loading docks, terminals, ports, or competent units [92].

### 4.2. Discharge-Type Ship Domestic Sewage Treatment Methods

#### 4.2.1. Conventional Domestic Sewage Treatment Methods for Ships

Discharge-type ship domestic sewage treatment methods refer to the treatment of domestic sewage generated by a ship through a series of technical means of equipment to meet the requirements of the IMO and the relevant regulations of the discharging area and then discharged directly into the environment of the outboard water body [93]. Large inland waterway transport vessels and marine transport vessels usually use domestic sewage treatment devices to treat domestic sewage to the required standard and then discharge it [37,94]. In the past, the commonly used domestic sewage treatment technology was mainly WCB-type, WCV-type, ST-type, AWW-type, and OMNIPURE-type devices as the representative of the ship domestic sewage treatment devices. These devices have the advantages of eliminating pollutants more thoroughly and do not cause secondary pollution of the environment but, due to the large size of the device and other shortcomings, were gradually outlawed [95].

#### 4.2.2. Advanced Ship Domestic Sewage Treatment Methods

##### Biotechnology-Based Domestic Sewage Treatment Methods for Ships

The high salt content of a ship’s sewage is recognized as a critical element affecting the efficiency of removal in ships’ sewage treatment systems [96]. Ren-Cang Li et al. showed that a high salt concentration (>21 g/L) negatively affected COD and NH_4_-N removal efficiency, whereas a low salt concentration (≤21 g/L) had a negligible effect [97]. For high-salinity ship domestic sewage, Yating Li, Yafei Wang et al. removed about 85.4% of total nitrogen (from 200 mg/L), 87.8% of total phosphorus (from 40 mg/L), and 98.6% of COD (from 1600 mg/L) from toilet-flushing effluent without additional carbon addition and O_2_ provision from a new system of marine bacterial algae [82]. Li Jiang et al. proposed a bioaugmentation strategy for the treatment of high-salinity black water using Pseudoalteromonassp.SCSE709-6. In serial incubation trials aboard a ship, systems that were inoculated with bio-enhanced bacterial strains showed excellent removal of COD (99.8 percent of maximum removal) and TP (88.39 percent of maximum removal), whereas TN could be improved further [98]. Xingxing Zhang et al. investigated a novel integrated landscape eco-treatment system (LIETS) for maintaining ship sewage treatment based on a modified denitrification phosphorus removal (mDPR) craft. The experimental results through 200 days showed that the removal efficiency of TN, TP, and COD reached 82.0%, 81.0%, and 94.0%, respectively, and the effluent concentration was 10.9 mg/L, 0.97 mg/L, and 17.8 mg/L, respectively [99].

##### MBR Technology-Based Domestic Sewage Treatment Methods for Ships

Existing research trends show that ship domestic sewage treatment is developing towards new treatment methods such as the membrane bioreactor (MBR). The research of combining MBR with other domestic sewage treatment methods will become a hot spot for ship sewage treatment in the future. A novel anaerobic micro-sludge membrane bioreactor for marine domestic wastewater treatment was proposed by Yuhang Cai et al. Their study showed that the novel aerobic–anaerobic micro-sludge membrane bioreactor (O-AMSMBR) had better COD and TN removal efficiencies (average COD removal = 91.6%, average TN removal = 88.07%); the anaerobic sludge volume in the O-AMSMBR was reduced by 80%, but the TN removal was 10% higher than in conventional membrane bioreactor (MBR) technology [100]. The effectiveness of a hybrid membrane bioreactor (HMBR) in degrading organic pollutants at high substrate concentrations in ships was demonstrated in a study by Linan Zhu et al. At a capacity of 2.4 kgCOD/(m^3^/day), the average COD removal rate of HMBR was as high as 95.13%, and the COD concentration in the effluent was 48.5 mg/L, which is far below the International Maritime Organization (IMO emission standard of 125 mg/L) [101]. In a study by Xin Li et al., a novel pilot-scale air-lift multistage recirculating membrane bioreactor (AMCMBR) was used to treat real ship sewage, and the results showed satisfactory COD and TN removal efficiencies (R_e(COD)_ 91.57% and 87.82%; Re_(TN)_ 77.17% and 81.19%, respectively) [102]. For high-salinity ship domestic sewage, studies have proved that MBR systems have better performance in treating ship’s domestic sewage with different salinities [103].

**Figure 4:**
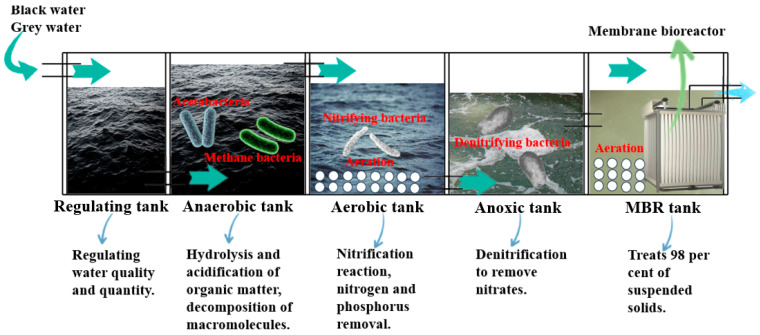
Schematic diagram of the main flow of the AOA-MBR process for ship domestic sewage treatment.

Based on the features of domestic sewage from inland navigation vessels, Yu, S.H. et al. developed a kind of micro-integrated device of the A/O + MBR process to deal with the ship’s domestic sewage, and the operation results show that the device has good treatment effects on a small ship’s domestic sewage [104]. Young-Ik Choi et al. developed a compact domestic sewage effluent treatment device by applying SBR and MBR processes and evaluated the usability of the device for the disposal of domestic sewage onboard ships, with an average removal of 91% of the heavy metal chromium, 93% of TN and 95% of TP in the experiment [105]. In a study by Daniela Piazzese et al., the MBR test device achieved about 99% removal of total petroleum hydrocarbons, confirming the robustness of the MBR technology for the treatment of ship domestic sewage [106]. Studies have shown that the MBR device configured on the ship can gradually remove pharmaceutical compounds (peak MBR discharge concentrations were 0.47 μg/L for carbamazepine, 6.8 μg/L for ibuprofen, and 0.3 μg/L for diclofenac), demonstrating the purification effect of the MBR device on pharmaceutical compounds [44]. There are also studies on the coupling of various sewage treatment technologies to construct a new type of full-scale oxic–anoxic micro-sludge membrane biofilm reactor for marine domestic sewage treatment, which has a stable sewage treatment efficiency and achieved chemical oxygen demand (COD) and total nitrogen (TN) removal rates of more than 82% and 76% in ship’s domestic sewage [107]. Yuan, M. adopts the external ultrafiltration membrane of anaerobic/aerobic/anoxic alternating operation of the sequential batch activated sludge method (AOA-MBR) synchronous denitrification and phosphorus removal in a new process for the treatment of a ship’s domestic sewage, under the premise that the influent TP is lower than 30 mg/L and the TP removal rate can be up to 95%, which shows that the AOA-MBR reactor is especially suitable for the treatment of ship’s domestic sewage, which has a low C/P ratio, low C/N ratio, and large changes in the quality of water, and it can solve the unstable problem of ship’s domestic sewage [108]. The AOA-MBR simultaneous denitrification and phosphorus removal process consist of an anaerobic, aerobic, and anoxic three-time cycle MBR tank and a continuously operating membrane module, and the membranes used are ultrafiltration membranes. A process flow diagram of AOA-MBR for ship domestic sewage treatment is shown in Figure 4.

Liu, Y.Y. designed a set of improved membrane bioreactors, in front of the traditional membrane bioreactor; the original conditioning pool in the process equipment was converted into an anoxic pool and used for cultivating anoxic microorganisms, and the original primary sedimentation pool was converted into a water storage pool for storing incoming water and part of the outgoing water to ensure that the circulation of the whole equipment is carried out normally. The original water tank was converted into a drainage tank, which was used to collect sewage and sludge from each pool after the experiment to unify the treatment and prevent secondary pollution. The quality of the effluent meets the discharge standard [109]. In a study by Zhang Zhiliu, et al., aerobic particulate silt was cultivated in MBR to treat ship domestic sewage, and the experimental results indicated that when ρ(Al^3+^) = 30 mg/L, aeration rate = 0.8 m^3^/h, and sludge settling time = 30 min, aerobic particulate sludge was formed in the shortest time (30 days); the removal rates of TP, TN, and COD in the reactor were 92.37%, 92.37%, 93.46%, and 97.39%, respectively, 93.46% and 97.39%, and the membrane contamination was improved [110]. Wang Tianqi used magnetic flocculation-coupled IAMBR to treat ship domestic sewage, and the experimental findings indicated that the magnetic flocculation-coupled IAMBR reactor has a better effect on the treatment of ship domestic sewage; the addition of polymerized aluminum chloride in the reactor significantly improved the removal effect of TP. The average removal rates of COD_Cr_, ammonia, TN, and TP were 96.46%, 83.24%, 86.21%, and 94.74%, and the effluent concentrations of COD_Cr_, ammonia, TN and TP in the reactor were lower than 30 mg/L, 8 mg/L, 9 mg/L and 0.5 mg/L, respectively, which can reach the latest discharge standard of domestic sewage [111]. Some of the new ship domestic sewage treatment methods are shown in Table 5.

## 5. Conclusions and Outlook

Ships generate domestic sewage in the course of their operation, which causes serious contamination of water resources and damage to the water environment of rivers, lakes, and seas. Domestic sewage discharged from ships contains high concentrations of nitrogen, phosphorus, and other nutrients, which can lead to eutrophication of the aquatic environment and disrupt the self-purification balance of the waters, while the release of viruses, bacteria, and parasites can endanger the hygiene and health standards of the waters. Domestic sewage discharged by ships contains a mixture of several hundreds of petroleum hydrocarbons, which adversely impacts the health and ecology of aquatic organisms. In addition, domestic sewage discharged from ships may contain microplastics, pharmaceutical compounds, and toxic and hazardous substances, such as organotins and heavy metals, which are harmful to marine mammals and aquatic organisms. The discharge of domestic sewage from inland waterway vessels also poses a serious hazard to the environment of the surrounding waters and the health of residents. Therefore, ship domestic sewage treatment is crucial. Ship domestic sewage treatment methods are mainly divided into two types: discharging type and non-discharging type. Non-discharge-type treatment includes methods such as sewage collection devices, which are applicable to the situation where the ship does not discharge sewage into the water environment during navigation. In contrast, discharge-type treatment means that the ship’s domestic sewage is treated by the equipment and then discharged directly into the water environment. Traditional domestic sewage treatment technologies include contact oxidation (WCB-type), WCV-type, activated sludge (ST-type), AWW-type, and OMNIPURE-type devices, but they have gradually been outlawed due to the disadvantages of larger size, lower reliability, and higher equipment failure rate. In recent years, there has been tremendous development of novel treatment methods, such as membrane bioreactor (MBR) systems, with better COD and TN removal efficiency. The emergence of these novel technologies offers new possibilities to improve the quality of ship sewage treatment and helps to reduce the pollution of water resources from ships. The shipping industry’s growth has made ship sewage treatment increasingly urgent. MBR technology dominates this field, with most research aiming to enhance its effectiveness. However, there is a need to explore integrating MBR with other treatment methods or developing new ones. Despite its advantages like small size and high-quality effluent, MBR faces issues like membrane contamination, which can cause operational challenges and increased costs, indicating areas that require future research focus.

## Figures and Tables

**Table 1:** Ship and city domestic sewage comparison.

Characteristics	Ship’s Domestic Sewage	Reference	City Domestic Sewage	Reference
NH4^+^-N	800~1300 mg/L	[10]	8.1–49.3 mg/L	[11]
Salinity	6500 ± 300 mg/L	[12]	340–600 mg/L	[13]
Ammonia	1250 ± 250 mg/L	[12]	8–25 mg/L	[13]
Phosphate	190 ± 10 mg/L	[12]	15.3 ± 1.2 mg/L	[11]
COD	7067 ± 470 mg/L	[12]	537.0 ± 48.7 mg/L	[11]
Microplastic	72 n/L	[14]	32.5 ± 1.0 n/L	[15]
TSS	5000 mg/L	[16]	388.0–597.5 mg/L	[11]
BOD_5_	550 mg/L	[17]	274.6–397.7 mg/L	[11]
Total *Coliform bacteria*	315 CFU/100 mL	[17]	6.47 ± 0.75 CFU/100 mL	[11]

**Table 2:** Comparison of various pharmaceutical compounds and their concentrations in municipal and ship’s domestic sewage [44].

Pharmaceutical Compounds	Concentration in Urban Sewage	Concentration in Black Water	Concentration in Gray Water
Carbamazepine	0.18–0.19 μg/L [46]	3.9 μg/L	0.05–0.06 μg/L
Lbuprofen	6.4–9.4 μg/L [46]	29 μg/L	8.5–9.2 μg/L
Diclofenac	4.2 μg/ML [47]	0.04 μg/L	0.65 μg/L
Propyphenazone	<6.05 μg/L [48]	1.0 μg/L	0.33–0.38 μg/L
Metoprolol	-	71.5–80.0 μg/L	8.2–8.5 μg/L
Bezafibrate	-	6.3–7.4 μg/L	0.41–0.47 μg/L
Clofibric acid	-	0 μg/L	0.05 μg/L
Clarithromycin	1.86–4.47 μg/L [49]	6.5–7.3 μg/L	0.025–0.03 μg/L
Sulfamethoxazole	16–118 ng/L [50]	0.69–0.75 μg/L	0.1 μg/L
Trimethoprim	17.0–39.5 ng/L [51]	9.5–9.7 μg/L	0.98 μg/L
Benzophenone	-	7.5–7.7 μg/L	1.0 μg/L
Atenolol	85.9–206 ng/L [51]	0.71–0.74 μg/L	0 μg/L
Tonalide	-	0.1 μg/L	1.0 μg/L
Caffeine	225 μg/L [48]	29.1–30.2 μg/L	210–260 μg/L
Bisphenol A	-	29.1–30.2 μg/L	8.0 μg/L
TCPP	-	7.10–7.28 μg/L	170–205 μg/L

**Table 3:** Comparison of IMO and Chinese standards for domestic sewage discharge from ships.

Restricted Indicators	IMO				GB3552-2018 ^a^		
	MEPC.2(IV)	MEPC.159(55)	MEPC.227(64)	MEPC.227(64) Special Area Passenger Ships	GB 3552-2018 Before 1 January 2012	GB 3552-2018 1 January 2012 and Onwards	GB 3552-2018 River Passenger Ships from 1 January 2012 and Onwards
PH	6–9	6–8.5	6–8.5	6–8.5	-	6–8.5	6–8.5
BOD_5_ (mg/L)	50	25	25	25	50	25	20
COD (mg/L)	Not required	125	125	125	-	125	60
Number of heat-resistant E. coli groups (CFU/100 mL)	250	100	100	100	250	100	100
TSS (mg/L)	50	35	35	35	150	35	20
Residual chlorine (mg/L)	As low as possible	<0.5	<0.5	<0.5	-	<0.5	<0.5
NH_4_-N (mg/L)	-	-	-	-	-	-	15
TN (mg/L)	-	-	-	20 (mg/L) or at least 70 percent reduction	-	-	20
TP (mg/L)	-	-	-	1.0 (mg/L) or at least 70 percent reduction	-	-	1.0

^a^ Apply to inland rivers and seas within 3 nautical miles from the nearest land.

**Table 4:** Difficulties in treating domestic sewage from ships and their characteristics.

Difficulties	Characterizations	Reference
Ship area constraints	Compact space on board restricts the footprint of the sewage treatment unit from being too large, and a small treatment unit may result in substandard sewage treatment.	[80]
The high salinity of sewage	Ocean-going ships use seawater to flush toilets directly, resulting in sewage salinity much higher than normal, and high salinity sewage will have an impact on the microorganisms in the biochemical method sewage treatment device, reducing its treatment effect and efficiency.	[81,82]
Solution of sludge after sewage treatment	When treating sewage, if the sludge after sewage treatment is not taken into account, it will lead to excessive accumulation of sludge in the sewage treatment system, which in turn will lead to a reduction in sewage treatment efficiency.	[72]
Problems with the power supply to the sewage treatment	The increasing capacity of the ship’s electrical load may lead to the emergence of a tight power supply. At this time, the power consumption of the sewage treatment device must be controlled within the loadable range of the ship’s power supply system to ensure the normal operation of the ship.	[83]
Poor stability of sewage	Ships often rock during navigation, which affects the operation of the treatment device, and at the same time, the concentration of sewage entering the treatment device varies greatly, which may cause the effluent water quality to fail to meet the standards.	[84,85]
Mixed types of pollutants	In addition to conventional pollutants, domestic sewage from ships often contains heavy metals, pharmaceutical compounds, microplastics, a variety of organic compounds, etc. The pollutants are of various types, and the treatment surface of the treatment device is highly required.	[86]
High pollutant concentration	Ships’ water conservation measures have led to high concentrations of various types of pollutants in their lives, with the concentrations of many kinds of pollutants being more than ten times that of normal conditions.	[16,87]
Sludge is highly toxic	Sludge consists of a wide range of hazardous substances, such as dioxins and polychlorinated biphenyls (PCBs), which are difficult to treat and expensive to reuse.	[88]

**Table 5:** Novel ship domestic sewage treatment methods.

Treatments	Treatment Effect	Characteristics	Reference
Marine Bacterial Algal Systems	Removal of about 85.4% TN, 87.8% TP, and 98.6% COD.	No additional carbon addition and O_2_ supply are required	[82]
Pseudoalteromonassp.SCSE709-6	COD (maximum removal of 99.8%), and TP (maximum removal of 88.39%).	Inoculated with bio-enhanced strains	[98]
LIETS	Removal efficiencies of 82.0%, 81.0%, and 94.0% were achieved for TN, TP and COD, respectively.	Based on the modified denitrification phosphorus removal (mDPR) process	[99]
O-AMSMBR	Average COD removal = 91.6% and average TN removal = 88.07%.	Based on the MBR process, has lower sludge volume, higher TN removal rate	[100]
HMBR	The average COD removal was up to 95.13%.	Based on the MBR process	[101]
AMCMBR	The average removal rate of COD was 91.57 percent and 87.82 percent, respectively; the average removal rate of TN was 77.17 percent and 81.19 percent, respectively.	Based on the MBR process	[102]
SBR + MBR	The average removal rate of Cr is 91%, the average removal rate of TN is 93%, and the average removal rate of TP is 95%.	Based on the MBR process	[105]
AOA-MBR	The TP removal rate can go up to 95 percent.	Suitable for sewage treatment with low C/P ratio, low C/N ratio, and wide variation of water quality.	[108]
MBR + aerobic granular sludge	TP, TN, and COD removal rates were 92.37, 93.46, and 97.39 percent, respectively.	Cultivate aerobic granular sludge in MBR.	[110]
IAMBR	The average removal rates of COD_Cr_, NH4-N, TN, and TP were 96.46 percent, 83.24 percent, 86.21 percent, and 94.74 percent, respectively.	Addition of polymerized aluminum chloride (PAC) to the reactor	[111]

## Data Availability

The study did not report any data.
